# Recombinant cold shock domain containing protein is a potential antigen to detect specific antibody during early and late infections of *Haemonchus contortus* in goat

**DOI:** 10.1186/s12917-020-2261-6

**Published:** 2020-02-03

**Authors:** Muhammad Ali-ul-Husnain Naqvi, Kalibixiati Aimulajiang, Muhammad Ali Memon, Muhammad Waqqas Hasan, Sana Zahra Naqvi, Shakeel Ahmed Lakho, Wen Chu, Lixin Xu, Xiaokai Song, Xiangrui Li, Ruofeng Yan

**Affiliations:** MOE Joint International Research Laboratory of Animal Health and Food Safety, College of Veterinary Medicine, Nanjing Agricultural University, No.1, Weigang, Nanjing, Jiangsu Province, People’s Republic of China 210095

**Keywords:** *Haemonchus contortus*, Early diagnosis, Cold shock domain containing protein, Immunoblotting, Indirect-ELISA

## Abstract

**Background:**

*Haemonchus contortus* (*H. contortus*) is one of the most important parasites that cause huge economic losses to small ruminant industry worldwide. Effective prognosis and treatment depend upon the early diagnosis of *H. contortus* infection. To date, no widely-approved methods for the identification of prepatent *H. contortus* infection are available to identify prepatent *H. contortus* infection properly. The aim of this study was to evaluate the diagnostic potential of recombinant cold shock *H. contortus* protein (rHc-CS) during early and late infections of *H. contortus* in goat.

**Results:**

Purified rHc-CS exhibited a clear band, with a molecular weight about 38 kDa. *H. contortus* eggs were not detected by fecal egg count technique from feces collected at 0 to 14 days post infection (D.P.I). However, eggs were detected at 21, 28 and 35 D.P.I. Hence, results of immunoblotting assay showed specific anti rHc-CS antibody detection in all goat sera collected at early stage (14 D.P.I) and late stage (21–103 D.P.I) of *H. contortus* infection. Furthermore, no cross reactivity was observed against *Trichinella spiralis*, *Fasciola hepatica* and *Toxoplasma gondii* or uninfected goats. Among several evaluated rHc-CS indirect-ELISA format variables, favorable antigen coating concentration was found 0.28 μg/well at 37 °C 1 h and overnight at 4 °C. Moreover, optimum dilution ratio of serum and rabbit anti-goat IgG was recorded as 1:100 and 1:4000, respectively. The best blocking buffer was 5% Bovine Serum Albumin (BSA) while the best time for blocking, serum incubation and TMB reaction were recorded as 60, 120 and 10 min, respectively. The cut-off value for positive and negative interpretation was determined as 0.352 (OD_450_). The diagnostic specificity and sensitivity of the rHc-CS, both were recorded as 100%.

**Conclusion:**

These results validated that rHc-CS is a potential immunodiagnostic antigen to detect the specific antibodies during early and late *H. contortus* infections in goat.

## Background

*Haemonchus contortus* (*H. contortus*) is an important haematophagous gastrointestinal parasite of small ruminants. It can suck about 0.05 mL blood per day [1, 2] and may cause acute anemia, edema, diarrhea, severe frailty weight loss, and ultimately death [3, 4]. Due to high mortality and morbidity rate, *H. contortus* infection causes significant economic losses to small ruminants particularly in humid, tropical and subtropical regions [5, 6]. China mainly contributes 17.3% of world’s total goat population [7] in which different prevalence rate of *H. contortus* infection has been reported in several provinces [8]. The control of this parasite mainly relies on accurate and early diagnosis. Conventional fecal egg counts technique is main method to diagnose this infection clinically but it is difficult to detect *H. contortus* eggs in feces before 21–25 days of infection [4]. Last larval stages of this parasite feed on blood [9] and may suck up to 1/5th of total circulating erythrocyte volume in young animal [10]. *H. contortus* blood feeding starts at 11th day of infection [11] but clinical signs usually become apparent when infection becomes severe [12]. Another way for the diagnosis of this infection depends on the degree of anemia using FAMACHA system in which an ocular conjunctiva color chart is used for assessment of anemia to decide which animal requires treatment for *H. contortus* infection [13]. However, these methods are often nonspecific, insensitive, laborious, time consuming [14] and most importantly lacking the ability to detect the infection at early stage. Hence, early detection of *H. contortus* is crucial and necessary to control infection effectively [15].

During early infection, parasites produce and release Excretory and Secretory Products (ESPs) that play an important immunological role [16]. ESPs have been widely used as diagnostic antigen because these products have good specificity and sensitivity [17]. ESPs contain numerous proteins which depress the immunity of host at prepatent stages of infection by modulating immune system [18]. Recently, immunoblotting and ELISA based on different types of antigens (somatic and crude) have been reported for the detection of *H. contortus* specific antibodies [4, 15, 19, 20]. However, shared antigenic composition is major disadvantage of these antigens that leads to cross-reactivity in the diagnosis of *H. contortus* infection [21]. Currently, there is a lack of potential immunogenic antigen which can accurately detect the particular infectious stage of this helminth in goat. To overcome these challenges and to improve control strategies, a potential antigen based immunodiagnostic assay is needed [15].

Cold shock domain is present in every cellular compartment and it is a constituent part of nearly all prokaryotes and eukaryotes. In animals, cold shock proteins exhibit broad functions that relate to the growth and development of a cell. These proteins have special ability to bind with nucleic acid to regulate not only their own expression but also involve in the regulation of virulent genes [22]. In our previous proteomic study, interaction of *H. contortus* (Hc)ESPs with host peripheral blood cells at different developmental stages was reported. The Cold Shock domain containing protein (CS) is one of these HcESPs, that binds to goat PBMCs at L_4_ and L_5_ *H. contortus* development stages [23]. Hence the presence of CS protein may serve for diagnostic purposes as biomarker [24]. Thus, these proteins can perfectly act as immunodiagnostic antigen [17] to detect *H. contortus* infection at early stage.

This study was designed to evaluate the diagnostic capacity of recombinant cold shock *H. contortus* protein (rHc-CS) and to detect specific antibodies during early and late *H. contortus* infections in goat using immunodiagnostic assays.

## Results

### Purification, immunoblotting and early diagnostic potential

**Fig. 1:**
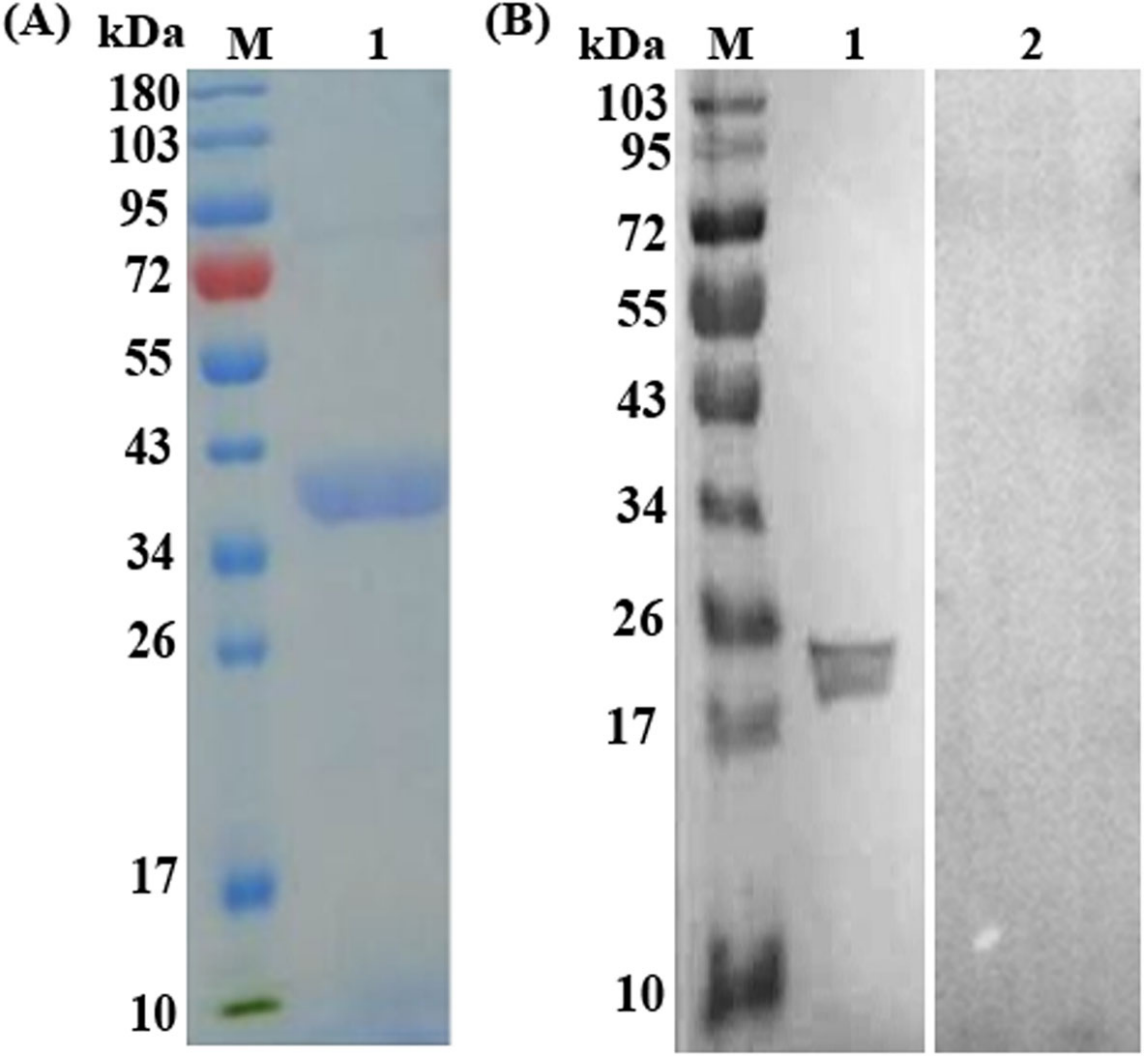
Purification and immunoblotting of rHc-CS protein. Lane M: standard protein pre stain molecular weight Marker. **a** Lane 1: Purified rHc-CS protein. **b** Lane 1: HcESPs were detected by rat anti-rHC-CS protein antibodies; Lane 2: membrane incubated with normal rat sera (as control)

**Fig. 2:**
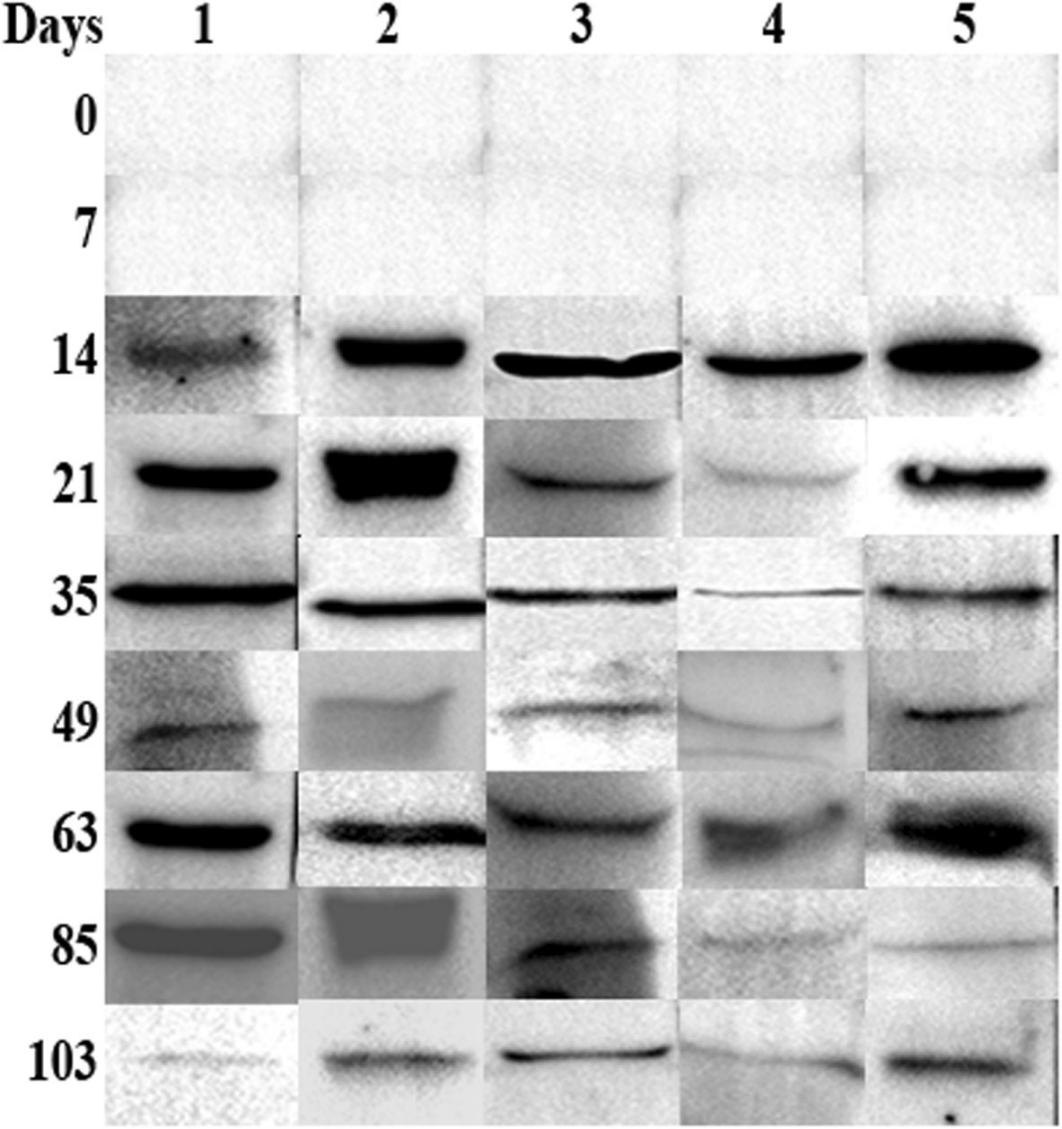
Immunoblotting of rHc-CS for specific antibody detection during different stages of *H. contortus* infections. Horizontal axis: Lanes 1–5: Membrane incubated with sera of five representative goats (Group 1) infected with 8000 *H. contortus* L_3_, Vertical axis: Different days of serum collection (0, 7, 14, 21, 35, 49, 63, 85, and 103). Antibodies against rHc-CS were detectable between 14 and 103 days post infection, but not on day 0 or 7

**Fig. 3:**
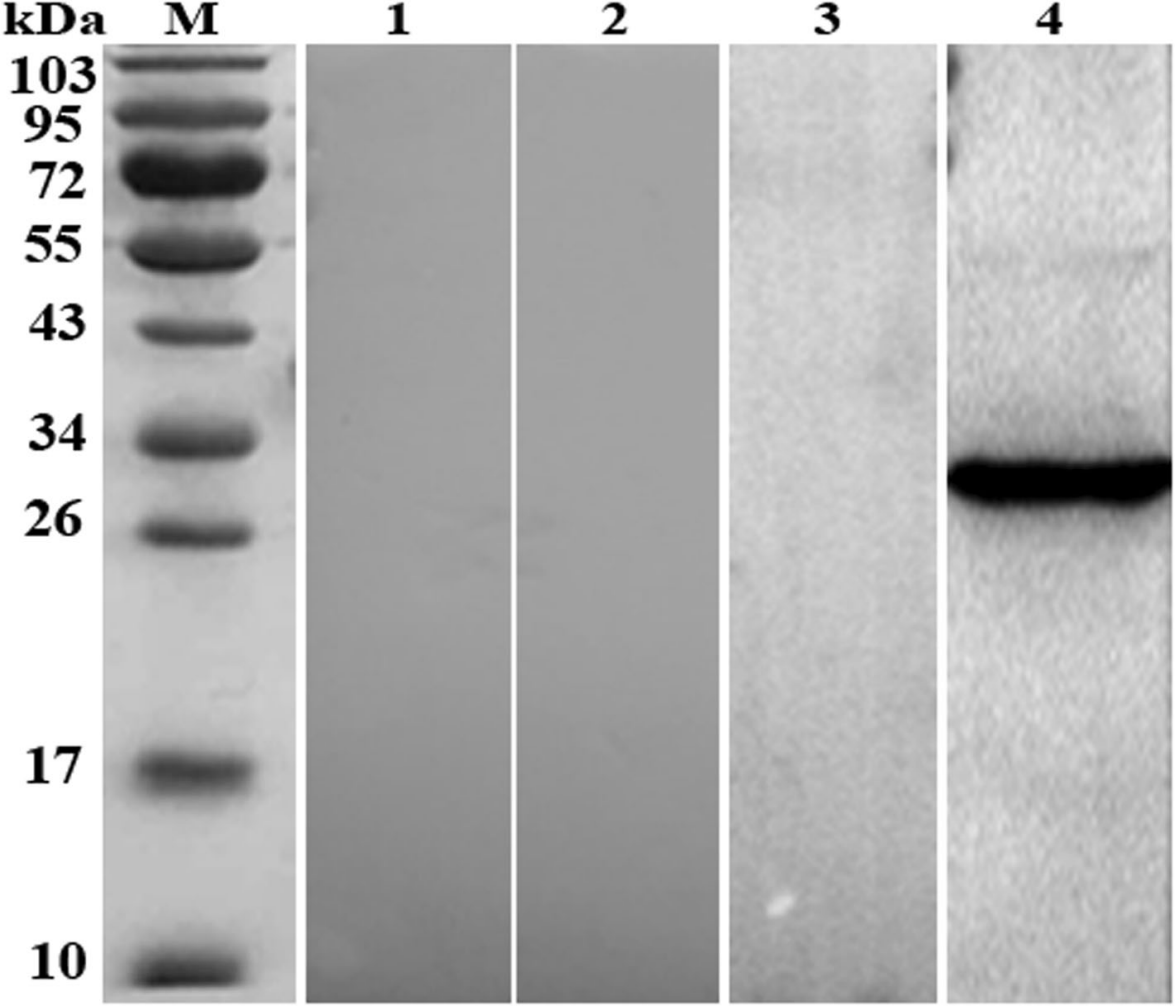
Immunoblotting of rHc-CS antigen to determine cross reactivity. Lanes shown are as follows: M = standard protein pre stain molecular weight Marker; Lane 1: membrane incubated with positive *T. spiralis* serum; Lane 2: membrane incubated with positive *F. hepatica* serum; Lane 3: membrane incubated with positive *T. gondii* serum; Lane 4: rHc-CS was recognized by goat anti-*H. contortus* sera (positive control)

The rHc-Cs was purified as Histidine-tagged fusion protein and resolved on 12% SDS-PAGE which showed single band of about 38 kDa (Fig. 1a). Immunoblotting results demonstrated that HcESPs could be recognized by anti- rHc-CS antibodies generated in Sprague Dawley (SD) rats. Furthermore, the native CS protein showed molecular mass of about 20 kDa (Fig. 1b, Lane 1) and no antibody was detected with untreated rat sera (Fig. 1b, Lane 2). Moreover, immunoblotting results showed that initial antibodies were detected in sera of all artificially infected goats (5/5) of group 1 collected at prepatent stage (14 D.P.I). The sera of all *H. contortus*-infected goats had detectable antigenicity at 21, 35, 49, 63, 85, and 103 days of infection, no IgG antibody against rHc-CS was detected in sera collected at day pre-infection and 7 D.P.I (Fig. 2). On the other hand, FECs showed that strongylid eggs were detected in all samples collected at 21 D.P.I (*mean* ± *SEM* = 7.4 ± 0.6), 28 D.P.I (*mean* ± *SEM* = 9.8 ± 0.78) and 35 D.P.I (*mean* ± *SEM* = 10.8 ± 1.08) but the fecal samples collected at 0, 7 and 14 D.P.I were negative for eggs. Furthermore, occurrence of *H. contortus* worms was confirmed at 30 D.P.I by necropsy in all tested goats. Moreover, the recombinant Hc-CS antigen did not show any cross-reactivity against goat sera infected with *T. spiralis*, *F. hepatica* and *T. gondii* (Fig. 3). These findings revealed that rHc-CS protein had good immune-reactivity and antigenic characteristics which detected the specific antibody during early and late stages of *H. contortus* infection. Conversely, *H. contortus* infection was not detected by microscopic examination at early stage (7 and 14 D.P.I).

### Optimization of rHc-CS indirect-ELISA

**Fig. 4:**
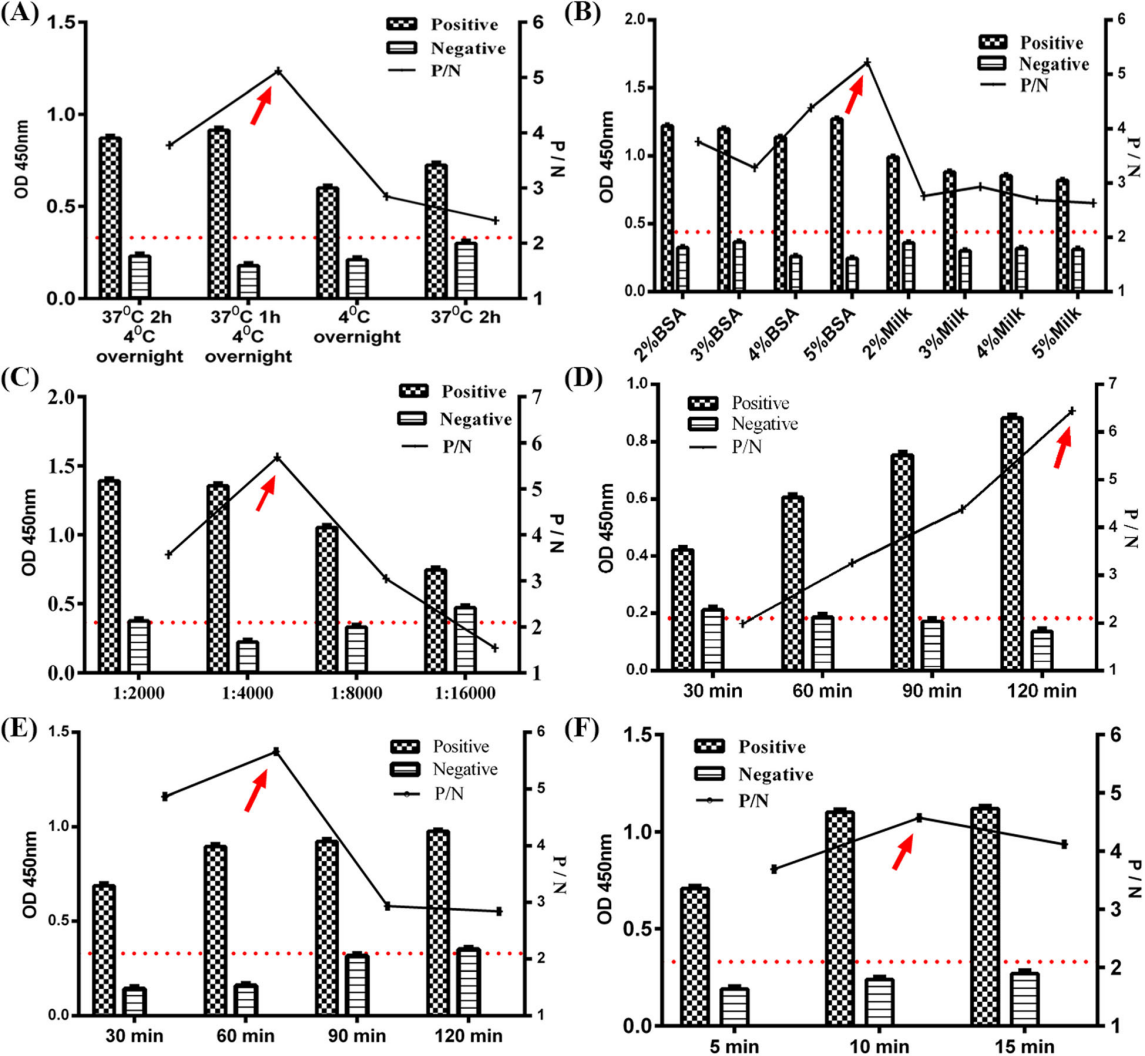
Optimization of indirect-ELISA by checkerboard titration. Positive bar: OD_450_ value of *H. contortus* infected goat sera; Negative bar: OD_450_ value of helminths frees goat sera; P/N: OD_450_ value of positive/ OD_450_ value of negative that showed on each bar and horizontal red dotted line is the cutoff line at 2.1 (right Y axis). P/N value more than 2.1 was determined to be positive while P/N value less than 2.1 was determined to be negative. The best condition (the highest P/N value) of each other was pointed by red arrows (↑). Graph **a**: Selection of antigen incubating condition; Graph **b**: Selection of blocking buffer; Graph **c**: Selection of optimal dilution of rabbit anti-goat IgG; Graph **d**: Selection of reactive time of serum; Graph **e**: Selection of blocking time; Graph **f**: Selection of reactive time of TMB. The optimum antigen incubating condition (37 °C 1 h and overnight at 4 °C), best blocking buffer (5% BSA), optimal dilution of rabbit anti-goat IgG (1:4000), best reactive time of serum (120 min), blocking (60 min) and reactive time of TMB (10 min) were observed

Several format variables were evaluated to optimize the indirect-ELISA based on rHc-CS, utilizing a small set of four samples containing two known *H. contortus* positive and two known negative sera (group 3). The optimum antigen (rHc-CS) coating concentration and the optimum dilution ratio of serum were 0.28 μg/well and 1:100 with the maximum P/N (OD_450_ = 5.085) value, respectively (Table 1). Subsequently, the optimum incubating condition, blocking buffer, secondary antibody dilution, serum incubation time and blocking buffer incubation time were recorded as 37 °C 1 h and overnight at 4 °C (P/N:5.117; Fig. 4a), 5% Bovine Serum Albumin (BSA; P/*N* = 5.222; Fig. 4b), 1:4000 (P/*N* = 5.706: Fig. 4c), 60 min (Fig. 4d) and 120 min (Fig. 4e), respectively. Moreover, the best Tetramethylbenzidine (TMB) reactive time was recorded as 10 min (Fig. 4f). However, this optimized rHc-CS indirect-ELISA was performed to determine the checkerboard titration of all sera that were already categorized as either *H. contortus* positive or negative.

**Table 1:** Determination of the optimal rHc-CS coating concentration and serum dilution for indirect-ELISA

Serum Dilution		OD_450_ values of rHc-CS at increasing coating concentrations						
		0.07	0.14	0.28	0.56	1.12	2.25	4.5
1:25	(P)	0.46	0.79	1.21	1.20	1.36	1.56	1.85
	(N)	0.19	0.30	0.35	0.53	0.64	0.94	1.61
	P/N	2.45	2.67	3.41	2.27	2.13	1.66	1.15
1:50	(P)	0.40	0.68	1.19	1.18	1.21	1.49	1.63
	(N)	0.18	0.21	0.29	0.34	0.55	0.77	1.41
	P/N	2.20	3.21	4.05	3.45	2.21	1.94	1.15
1:100	(P)	0.35	0.51	1.01	1.20	1.28	1.38	1.40
	(N)	0.17	0.17	0.20	0.26	0.44	0.68	1.29
	P/N	2.07	2.88	**5.08**	4.57	2.91	2.01	1.08
1:200	(P)	0.23	0.41	0.78	0.86	0.92	0.97	1.22
	(N)	0.17	0.13	0.16	0.22	0.30	0.50	1.00
	P/N	1.36	3.08	4.69	3.82	3.02	1.92	1.22

*Note*: Bold represent the optimum conditions for this indirect-ELISA method, the highest P/N value is 5.08

*Abbreviations*: *P* Positive serum, *N* Negative serum

### Determination of the cut-off value

To calculate the cut off value for negative sera in indirect ELISA, mean value (0.244 ± 0.004) was added into standard deviations (0.036) of OD values after multiplying by 3 as 3 × 0.036. OD values were obtained from 35 confirmed negative goat sera. The sera isolated from positive group showed significantly higher OD_450_ (> 0.352) as compared to negative group (OD < 0.352). One way ANOVA was performed to observe significant differences (*P* < 0.05) in mean OD values among infected goats’ sera by *H. contortus* (0.645 ± 0.012) and uninfected goats’ sera (0.244 ± 0.004).

### Receiver operating characteristic (ROC)

The influence of different cut-off values on the sensitivity and specificity of this antigen was investigated by ROC curve analysis. ROC analysis showed 100% sensitivity and specificity at cut-off value of 0.352. Moreover, least difference was seemed in sensitivity (96.8%) at cut-off value of 0.387 and specificity (96.8%) at cut-off value of 0.316. The area under the ROC curve (AUC) was recorded as 1 (*P* < 0.001) with the cut-off value of 0.352, which indicates high accuracy of the cut-off for classifying the serum samples into *H. contortus* positive or negative. Thus, the cut-off value for rHc-CS indirect-ELISA was selected as ≥0.352 for further use to provide maximum sensitivity with very good specificity.

### Sero-diagnostic potential of rHc-CS indirect-ELISA

**Fig. 5:**
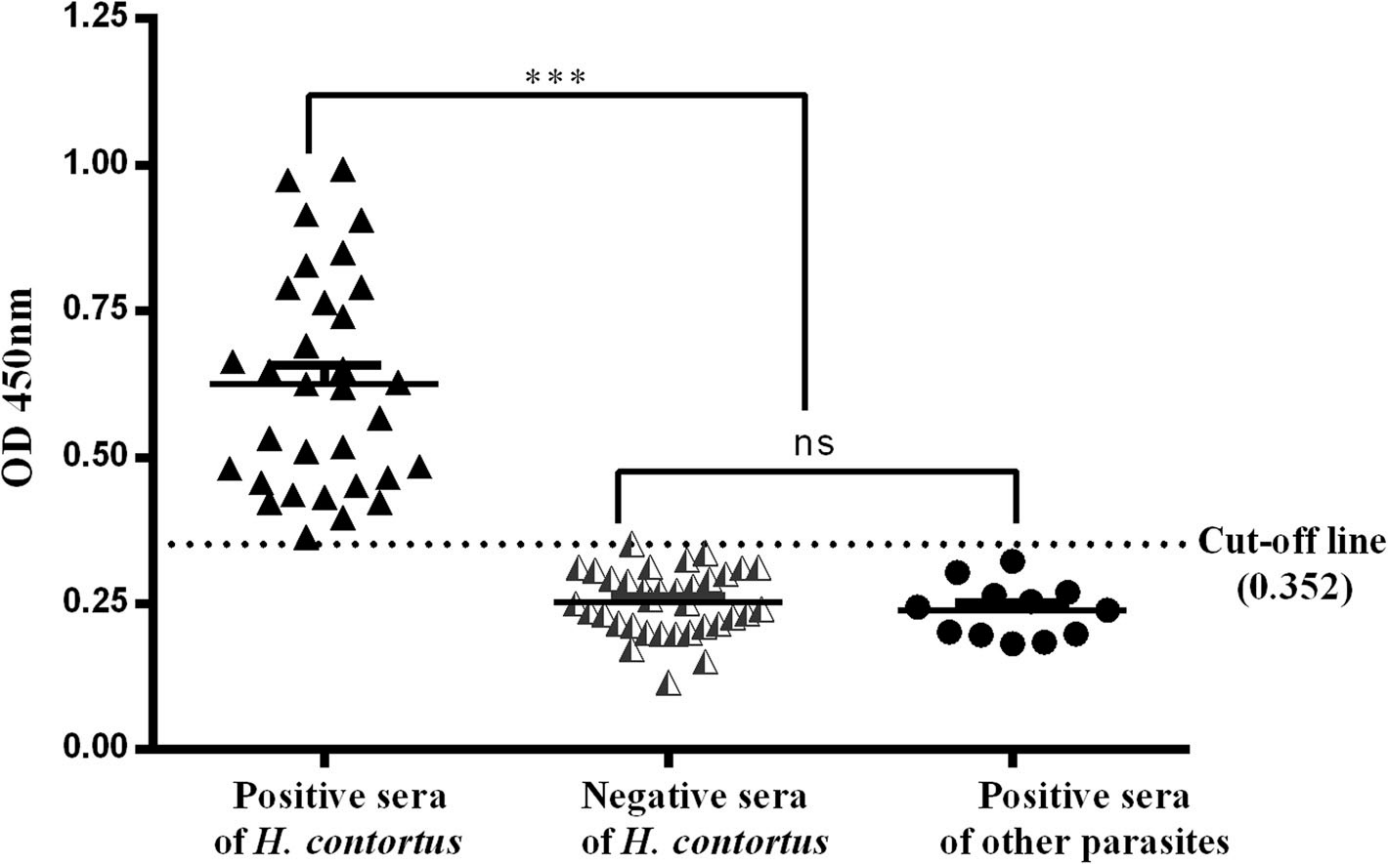
Sensitivity, specificity and cross-reactivity of indirect-ELISA based on rHc-CS. The dotted horizontal line represents the cut-off value (OD_450_ = 0.352) of indirect ELISA based on recombinant Hc-CS. Statistically significant differences (*P* < 0.001) were observed between *H. contortus*- positive sera and the other sera (*T. spiralis, F. hepatica* and *T. gondii*-positive and *H. contortus*-negative sera). No significant difference (*P* > 0.05) was noted between the *H. contortus*-negative and other parasites-positive serum samples

The rHc-CS has definite diagnostic potential through the optimized indirect ELISA. The indirect-ELISA based on rHc-CS showed 100% sensitivity against *H. contortus* positive sera while 100% specificity against untreated goat sera. Furthermore, no false positive/negative results were observed. Total 33 known positive sera were analyzed to evaluate the analytical sensitivity and rHc-CS indirect-ELISA showed 100% sensitivity as all positive sera (33/33) were above the cut-off line (0.352). The analytical specificity was evaluated using sera positive for *T. spiralis* (*n* = 4), *F. hepatica* (*n* = 4) and *T. gondii* (*n* = 4). Results of rHc-CS indirect-ELISA revealed that all sera positive for *T. spiralis*, *F. hepatica*, *T. gondii* and non-infected sera showed OD_450_ below the cut-off line. Scatter plot analysis showed significant differences (*P* < 0.001) between *H. contortus* infected sera and other parasites positive sera, while no significant difference (*P* > 0.05) was observed between helminths-free sera and other parasites’ positive sera (Fig. 5). In order to diagnose haemonchosis in goats, 100% sensitivity of this antigen was measured and no negative control was higher than the cut-off point.

## Discussion

*H. contortus* causes high mortality and morbidity rates in sheep and goats [5, 6]. To control this infection efficiently, early diagnosis of the infection is inevitable as last two larval stages of this parasite feed on blood that loss up to one fifth of the total circulating erythrocyte volume from young animals and may cause fatal anemia [10]. Previous study reported the serological methods for diagnosis of *Dicrocoelium dendriticium* because these methods are more accurate in comparison to FEC test [25].

Recently, larval antigen, somatic antigens and crude antigen have been evaluated for the immunodiagnosis of *H. contortus* infection [20, 26, 27]. However, the specificity and sensitivity is compromised for these antigens. ESPs are released by parasites within the body of host that play vital role in pathogenesis and modulation of the immune response at the early stage of infection. It is further added that *H. contortus* interaction with host starts after the transition of L_3_ into L_4_ stage [28]. Furthermore, recombinant protein, having high immunoreactive concentration, may act as best immunodiagnostic antigen by achieving high sensitivity and specificity [29–36]. Recombinant Hc-CS is a major constituent of HcES proteins [23] which may have potential to diagnose *H. contortus* at early stage of infection. This study was designed to evaluate the specific antibodies detection during early and late infections of *H. contortus* using immunoblotting and indirect-ELISA based on rHc-CS in experimentally infected goat. In this study, rHc-CS was used as diagnostic antigen of *H. contortus* infection for detection of specific antibody during prepatent period and development of indirect ELISA as well.

Immunoblotting is a useful technique to detect specific pathogen and antigen potency immunodiagnostic studies [37]. A confirmatory test for immunoblotting-positive sera was required to overcome the possible false positive results that may affect the certain diagnosis of infection [17]. In this study, immunoblotting results indicated that rHc-CS showed high immunoreactivity against polyclonal antibodies generated in sera of SD rats. The ELISA is a powerful tool as it provides a less time consuming, easy and safe way to perform serological detection. Therefore the association between ELISA and immunoblotting techniques can be seen as a potent way to increase sensitivity for immunodiagnosis purposes [38]. Previously, a study was conducted for prepatent detection of specific *H. contortus* antibody, early and late patency of *H. contortus* infections using ELISA based on Hc26 and immunoblotting in sheep [37]. Another study was performed using recombinant protein recIgE1–2 to detect *H. contortus* infection in sheep sera collected at 2 to 4 weeks post infection in which immunoblotting assay showed cross-reactivity against sheep sera [39]. In contrast, the immunoblotting results of this study revealed that rHc-CS antigen specifically reacted with antibodies present in all *H. contortus* positive goat sera at 14 D.P.I and persisted until 103 D.P.I. However, no anti-rHc-CS antibodies were detected in uninfected sera collected at day before infection. Previously, crude Excretory-Secretory (ES) protein of *H. contortus*-based Dot-ELISA was performed using sera collected at 1–3 weeks post infection sera but cross reactivity of this antigen with other nematode and trematode was not checked [15]. In contrast to this study, *H. contortus* antigen showed cross-reactivity against *Fasciola hepatica* and *Moniezia expansa* [40]. In previous study, somatic and adult larval antigen showed cross-reactivity with closely-related *H. contortus* species and possibly other genera [9]. In this study, rHc-CS did not show any cross-reactivity against co-infecting parasites (*T. spiralis*, *F. hepatica* and *T. gondii*). These results proved that specific-antibody detection has more specific and sensitive diagnostic potential as compared to conventional microscopic techniques. Thus, the evaluated rHc-CS can be used as diagnostic antigen to detect specific antibodies effectively during prepatent period of *H. contortus* infection in goat. Antigenicity, sensitivity and specificity may be influenced by the secretion time of antigen. It has been reported earlier that rHc-CS releases at 14 and 60 D.P.I and antibodies against this molecule are produced [23]. Another possibe reason might be the type of antigen used in study.

Indirect ELISA based on rHc-CS was optimized and developed to complement the immunoblotting results. Previously, combined use of ELISA and immunoblotting has been reported as a powerful tool to immuno-diagnose different infections [38, 41]. In this study, rHc-CS indirect-ELISA showed higher diagnostic specificity and sensitivity as compared to somatic antigen-based indirect-ELISA format reported in previous study [20]. This contrast might be due to higher binding efficiency of rHc-CS antigen, as type of diagnostic antigens used in study may influence specificity, sensitivity and cross reactivity of assay [42]. In previous study, indirect ELISA using somatic and adult larval antigen results in relatively lower diagnostic specificity [9]. In present study, both sensitivity and specificity of the rHc-CS indirect-ELISA were measured as 100%, that is similar to the findings of previous study in which His-ES24-based ELISA was performed to detect specific antibodies in *H. contortus* infected sera of sheep [17]. In contrast, 90% [43] and 87% [41] diagnostic sensitivity of indirect ELISA was reported using different recombinant *H. contortus* tropomyosin antigens.

Various types of false negative and false positive reactions may influence the results, irrespective of antigens in indirect-ELISA [44]. To determine the background noise reactions, optimization of best antigen and antibodies dilution are crucial to increase the diagnostic potential of indirect ELISA. After standardization of rHc-CS based indirect-ELISA, checkerboard titration results showed best performance with optimal coating concentration of antigen (0.28 μg/well), best serum dilution (1:100) and best working concentrations of secondary antibody (rabbit anti-goat IgG; 1:4000). Type of blocking buffers and their incubating time may also influence the performance of assay and may encourage false positive or false negative reactions [45]. In this study, to minimize this challenge of false reactions, different dilutions of different blocking buffers were optimized with different incubating times. Incubation time of buffer has significant effects on the performance of the assay. Therefore, the optimized results showed that the best blocking buffer was 5% BSA which is same as the findings of previous study [46]. The optimum reactive time of blocking and TMB was recorded as 60 and 10 min, respectively. Furthermore, the best P/N value (6.44) was recorded when serum was incubated for 120 min. To reduce time, serum can be incubated for 60 min because P/N value was observed positive (P/*N* > 2.1). This optimized rHc-CS based indirect-ELISA will be more authenticated tool for identifying goats in the prepatent stage of *H. contortus* infection than other antigen-based assays. The results of this study will facilitate the low cost early serological diagnosis of large number of animals and possibly vaccine development against the infection of *H. contortus*. This assay has high sensitivity and specificity for downstream application in field studies. However, further study is required to assess the cross-reactivity of rHc-CS against other nematodes belonging to Trichostrongylidae family. Additionally, to fine tune this assay epidemiological surveillance studies with huge number of samples are required.

The rHc-CS antigen based indirect-ELISA will be pivotal tool for farmers to identify *H. contortus* infection even at early stage in goat. For this purpose, veterinarian will be called at farms to collect blood for further analyses. After 10 days, blood samples will be collected again from the goats with negative results for confirmation.

## Conclusions

Combine use of immunoblotting and indirect ELISA showed that rHc-CS is a specific, sensitive, and potential immunodiagnostic antigen which may consistently detect *H. contortus* antibodies in goats during early as well as late infection of *H. contortus*.

## Methods

### Ethical statement

All experimental protocols were approved by the Science and Technology Agency of Jiangsu Province (Approval ID: SYXK (SU) 2010–0005).

### Study population

Local crossbred goats (*n* = 35, age≃6 months) were bought from a farm in Xuyu city, Jiangsu, China and kept under controlled conditions in animal house of Nanjing Agricultural University (NAU). All goats were orally dewormed twice with Levamisole (8 mg/kg) at two weeks interval to remove natural parasitic infections. Fecal samples were collected from all goats twice a week and analyzed microscopically for helminth eggs. Helminthes free goats were utilized for further experiment, After 25 days of first deworming, goats were divided randomly into group 1 (*n* = 5), group 2 (*n* = 28) and group 3 (*n* = 2).

Female SD rats of 150 g body weight (*n* = 6) were obtained from the Experimental Animal Center of Jiangsu, PR China (Certified: SCXK 2008–0004). Rats were separated randomly into two groups, group 1 (*n* = 3) and group 2 (*n* = 3) to collect polyclonal antibodies. Rats were kept in sterilized room with free access of food and water. Anti-rHc-CS polyclonal antibodies were collected following the methods as described early [47]. Briefly, complete Freund’s adjuvant was equally mixed with rHc-CS protein (300 μg) and injected subcutaneously in SD rats of group 1. After 14 days, incomplete Freund’s adjuvant mixed equally with rHc-CS protein was injected twice with 1 week interval. Finally, SD rats were anesthetized with 25% isoflurane (inhaling anesthesia) after 1 week of the last dose by open drop method [48]. Group 2 was kept untreated as control. Rats were euthanized by head dislocation after collecting blood from eye to prepare sera.

### *H. contortus* infective larva (L_3_)

The *H. contortus* strain was maintained by serial passages in helminth-free goats, at MOE laboratory NAU. *H. contortus* (L_3_) used in these experiments were obtained from feces of infected goats using conventional method [49]. Briefly, feces from *H. contortus* infected-goat were collected, crushed, mixed with water and combined with vermiculite to keep mixture moist at room temperature. The pan was covered with aluminum foil having several holes to allow air flow. After 10 days, mixture was filtered through cheesecloth to collect larvae to examine microscopically that were preserved at 4 °C in penicillin G mixed with water until use.

### Experiment 1

First experiment was performed to assess early diagnostic potential of rHc-CS protein during different stages of *H. contortus* infection using immunoblotting assay. For this purpose, group 1 (*n* = 5) was artificially infected with 8000 infected larva of *H. contortus* (L_3_) orally and group 3 (*n* = 2) was kept uninfected as control. Serum samples were collected from group 1 (infected) and group 3 (uninfected) at specified days; one day prior challenging infection and 7 to 103 days post challenging infection with week interval for antibody detection.

Moreover, McMaster egg count technique was performed to examine the fecal samples collected at 7, 14, 21, 28 and 35 D.P.I as described previously [50].

### Experiment 2

Experiment 2 was performed to develop and optimize indirect ELISA based on rHc-CS. In this regard, Group 2 (*n* = 28) was orally infected with 8000 *H. contortus* L_3_ and fecal examination was performed to confirm *H. contortus* infection before collecting the sera. Moreover, at 30 D.P.I the goats of group 2 were euthanized by injecting sodium pentobarbital (> 150 mg/kg) intravenously [48]. Furthermore, necropsy was performed to confirm the presence of adult worms embedded in the abomasum.

### Purification of recombinant Hc-CS

Recombinant plasmid (pET-32a + rHc-CS, GenBank: CDJ84294.1) was provided by MOE joint international Research Laboratory, Preventive Veterinary Medicine, NAU and protein was purified by following standard protocol [47]. After transformation of recombinant plasmid into *E.coli* BL21 (DE3), product was cultured in ampicillin containing LB (Luria Bertani) medium. Ni2+ nitrilotriacetic acid column (GE Healthcare, USA) was used to purify recombinant protein following the manufacturer’s instructions. The quantification of rHc-CS protein was analyzed by Bradford method [51] and protein was detoxified using Toxin Eraser™ Endotoxin Removal kit (GeneScript, USA).

### Immunoblotting assay

Immunoblotting is a method of choice for *H. contortus* infection. It helps to select target protein for the diagnostic purposes as well as for the antigenic immunogenicity and immunoreactivity evaluation [52]. Immunoblotting was performed to evaluate the immunoreactivity of rHc-CS using rat sera following the methods as described in our previous study [41]. Moreover, immunoblotting analysis was also performed to evaluate the antigenic characteristics of rHc-CS at early stage as well as late stage of *H. contortus* infection in sera of experimentally infected goats (Group 1). Upon each blood sampling day, the whole immunoblotting practice was repeated whereas the primary antibody differed. All anti *H. contortus* sera taken from infected (Group 1) and uninfected goats (Group 3), on those sampling days were taken as the primary antibody. Moreover, the specificity of rHc-CS against infected sera of *Fasciola hepatica* (*F. hepatica*), *Trichinella spiralis* (*T. spiralis*) and *Toxoplasma gondii* (*T. gondii*) was also checked by immunoblotting.

### Optimization and development of indirect-ELISA

Indirect-ELISA was performed to assess the immunodiagnostic potential of rHc-CS [53]. Indirect-ELISA based on rHc-CS was optimized by evaluation of numerous format variables including working dilution of rHc-CS antigen (4.5 to 0.07 μg/well), serum samples (1:25 to 1:200 dilutions), secondary antibody HRP conjugated rabbit anti-goat IgG (1:2000 to 1:16000) and working time of antigen coating (37 °C 2 h-4 °C overnight, 37 °C 1 h- 4 °C overnight), blocking (30–120 min), serum incubation (30–120 min) and TMB reaction (5–15 min). Furthermore, best blocking buffer (2, 3, 4, 5% BSA and 2, 3, 4, 5% Milk) was also determined using same method. Hence, checkerboard titration was used to derive OD values and calculated as the positive to negative (P/N) ratio [54].

In order to evaluate the diagnostic potential and to complement the immunoblotting results, standardized indirect-ELISA was developed as described previously [41, 55]. Moreover, serum samples (*n* = 33) from infected goats were used to calculate diagnostic sensitivity and serum samples of all goats before artificial infection (*n* = 35) were used to calculate diagnostic specificity of rHc-CS based indirect ELISA using following formula [56].

$$ Sensitivity=\frac{True\ Positive}{True\ Positive+ False\ Negative} $$

$$ Specificity=\frac{True\ Negative}{True\ Negative+ False\ Positive} $$

Additionally, to confirm the cross-reactivity against commonly found parasites, a total of 12 goat serum samples (4 samples for each parasite, Kept in the MOE laboratory of NAU) against *F. hepatica*, *T. spiralis* and *T. gondii* were used. All the experiments were performed in duplicate.

### Determination of cut-off value

Sera (*n* = 35) collected from all goats before *H. contortus* infection were used to determine cut-off value. The cut-off value was calculated by taking “mean absorbance values of known negative sera + (3× standard deviation)” [57]. OD_450_ value of sera greater than cut-off value was considered as sero-positive and OD_450_ value below cut-off value was considered as sero-negative [34].

### Statistical analysis

ROC analysis was used to simulate the influence of different cut-off values on sensitivity and specificity of the test [38]. ROC curves were obtained using statistical software MedCalc (version 15; http://www.medcalc.be). Statistical analysis of data was assessed by using software (Graph Pad Prism™ v6 07).

## Data Availability

All data generated or analyzed during this study are included in this published article.

## Abbreviations

AEC: Animal Ethics Committee
BSA: Bovine serum albumin
D.P.I: Days post infection
HcESPs: Excretory and secretory products of *Haemonchus contortus*
HRP: Horseradish peroxidase
IPTG: Isopropyl-b-D-thiogalactopyranoside
kDa: Kilodalton
MW: Molecular weight
NAU: Nanjing Agricultural University
OD: Optical density
P/N: Positive to negative
PBMCs: Peripheral blood mononuclear cells
PVDF: Polyvinyl difluoride membrane
rHc-CS: Recombinant Cold Shock domain containing protein
ROC: Receiver-operating characteristics
SD: Sprague Dawley
SDS-PAGE: Sodium dodecyl sulfate-polyacrylamide gel electrophoresis
TBS-T: Tris-Buffered Saline containing 0.05% Tween 20
TMB: Tetramethylbenzidine
